# The quest for ligands and binding partners of atypical cadherin FAT1

**DOI:** 10.1016/j.tranon.2021.101097

**Published:** 2021-04-18

**Authors:** Khushboo Irshad, Nargis Malik, Manvi Arora, Yakhlesh Gupta, Subrata Sinha, Kunzang Chosdol

**Affiliations:** Department of Biochemistry, All India Institute of Medical Sciences, New Delhi, India

**Keywords:** Glypican, Fat1, Cancer, GPC3, glypican-3, TM, transmembrane domain, GAG, glycosaminoglycan, GPI, glycosylphosphatidylinositol, RTP, receptor tyrosine phosphatase, EGF, epidermal growth factor, Ab, antibody, TRAB, T cell-redirecting antibody, CAR-T cells, chimeric antigen receptor T cells

## Abstract

A recent study in *Scientific Reports* identified glypican-3 (GPC3) as a novel extracellular interacting protein for FAT1 in hepato-cellular carcinoma (HCC) cells. FAT1 is a large transmembrane atypical cadherin with limited knowledge existing about its binding partners. While in Drosophila, *dachsous* (*ds*), another transmembrane member of the cadherin superfamily, is known to function as FAT1 ligand, no ligand is known in mammals so far. The revelation of GPC3 as a potential binding partner of FAT1 extracellular domain unfolds an opportunity to study potential triggers of FAT1 signaling in cancers. Available inhibitors of GPC3 in various phases of clinical trials also present an attractive option to curb GPC3-FAT1 signaling in tumors that overexpress these proteins.

## Graphical abstract

**Figure:**
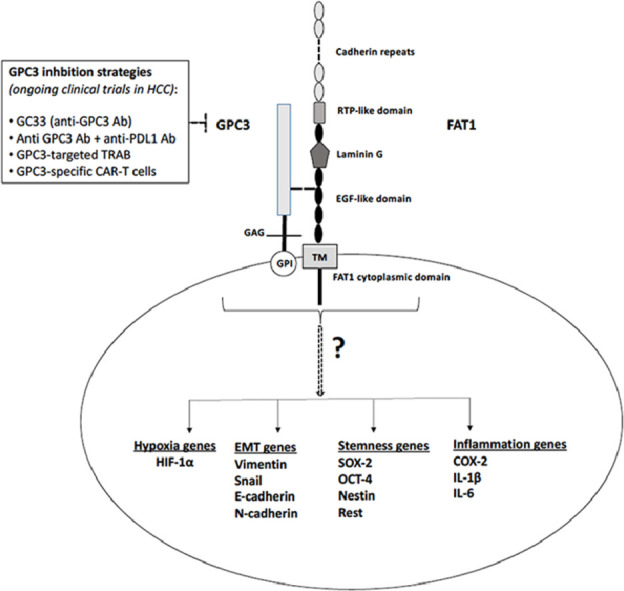

*FAT1*, the human homolog of Drosophila gene *fat*, has only been studied over the last decade for its role in embryonic development and cancers [1]. In Drosophila, *dachsous* (*ds*) plays the role of a putative ligand that binds with Fat protein to initiate signaling which controls planar cell polarity and organ growth during developmental stages [2]. However, in humans, a ligand that might trigger FAT1 signaling is yet to be identified. The only known interactions of FAT1 include those with clients like β-catenin which binds with its cytoplasmic domain, thereby affecting tumorigenic pathways [3]. We highlight here a recent study by Meng et al. which has identified glypican-3 (GPC3) as a novel protein that interacts with the extracellular domain of FAT1 in hepato-cellular carcinoma (HCC) cells [1].

GPC3 is a member of the heparan sulfate (HS) proteoglycan family [4]. It is attached to cell membrane via a glycosylphosphatidylinositol (GPI) anchor and is similar to other glypicans in having 14 conserved cysteine residues and HS side chains localized at C-terminus. Expression of glypicans is restricted to embryonic tissues and is undetected in adult liver. However, GPC3 shows elevated expression in HCC and contributes to tumor progression. Additionally, mechanistic studies have established the function of GPC3 as a co-receptor for Wnt proteins and other growth factors. In Drosophila, orthologues of GPC3 have been identified as *division abnormally delayed (Dally)* and *Dally-like protein (Dlp)* [2]. During normal organ development, these glypicans are regulated negatively by *dachsous* and *fat*; and function to adjust the extracellular distribution and signal efficiency of Wingless (Wg) ligand. Whether or not FAT1 regulates GPC3 expression during human embryonic development and cancers is a question that remains to be explored.

It is also pertinent to point out that previous literature has discussed the role of FAT1 in human cancers in two contexts- that of an oncogene as well as a tumor suppressor [5]. While FAT1 upregulation has been shown to promote cancers of liver, brain, breast, colon, and pancreas; mutations in FAT1 have been found to promote skin, lung, head and neck and oral cancers. Perhaps a deeper knowledge of the interplay between FAT1 and its associated molecules in different microenvironments might help in a better understanding of this tissue-specificity. However, the present article focuses on the oncogenic aspect of FAT1 in cancer.

GPC3 inhibition had been known to impede HCC cell proliferation via Yap inactivation. On the other hand, studies in Drosophila had also delineated a role of *fat* as a cell surface receptor in Yap signaling [1]. Given this connection between GPC3 and FAT1 in HCC development, Meng et al. sought to investigate a possibility of physical interaction between the two proteins which was revealed by co-immunoprecipitation (co-IP) analysis. Firstly, they annotated the structure of FAT1 protein downstream to cadherin repeats using SWISS-MODEL which predicted its similarity with extracellular domain of human receptor tyrosine phosphatase insulinoma-associated protein 2 (RTP-IA-2). In order to determine the region responsible for the observed interaction, truncated FAT1 fragments representing extracellular, transmembrane and intracellular domains were constructed and expressed along with full-length GPC3. The authors identified that only the extracellular region was able to retain the ability to co-immunoprecipitate GPC3. Smaller constructs of FAT1 extracellular domain and fine mapping by ELISA and flow cytometry were able to pinpoint the binding of GPC3 to the last four EGF-like domains (residues 4013-4181) in extracellular FAT1 region. The authors then compared the expression of GPC3 and FAT1 in normal liver samples and HCC cell lines where higher mRNA and protein levels were observed in HCC cell lines as compared to normal tissue. In addition, presence of hypoxia, which is known to aggravate solid tumors, was found to enhance the expression of GPC3 and FAT1 along with HIF-1α expression in HCC cells. Gene knockdown studies showed that individual as well as combined knockdown of GPC3 and FAT1 led to additive reduction in transwell migration of Hep3B cells and decreased the expression of epithelial-mesenchymal transition (EMT) markers Snail and Vimentin in hypoxic HepG2 cells, while increasing the expression of E-cadherin. Thus, while the study identifies GPC3 as a binding partner of FAT1 and presents GPC3-FAT1 as a functional complex, it opens new avenues to study underlying mechanisms that may connect FAT1 with tumor-related signaling pathways.

FAT1 is known to deregulate critical oncogenic signaling pathways. Our lab has reported FAT1 as a crucial regulator of glioma cell migration and invasiveness via upregulation of EMT genes like, Snail, Vimentin, LOX and N-cadherin [6,7]. Similarly, FAT1 was found to promote stemness and clonogenicity in glioma cells by regulation of stemness markers, such as SOX2, OCT4, Nestin and REST. Further, we showed that presence of hypoxia in the tumor microenvironment, which is a hallmark of glioblastoma, upregulates FAT1 expression. FAT1 knockdown in hypoxic cells led to reduction in HIF-1α expression, as a result of compromised EGFR-Akt signaling and increased VHL-dependent proteasomal degradation of HIF-1α [8]. Hence, accumulating evidence points to an important role of FAT1 in regulating various signaling pathways that operate during glioma pathogenesis. However, the exact underlying mechanism that drives the giant transmembrane FAT1 cadherin to impact transcription of these genes involved in cell migration, invasion, stemness and hypoxic response is yet to be deciphered. While it may be too early to confer the role of transcription factor to FAT1 for upregulation of EMT and stemness genes, it would now be fascinating to study the impact of GPC3 (and other GPC isoforms) interaction on FAT1 signaling event that initiates at the cell membrane of tumor cells. Hence, a deeper delve into GPC3-FAT1 interaction is expected to address the missing link between FAT1 expression level and the molecular mechanisms that drive mesenchymal phenotype of tumor cells (Fig. 1).

**Fig. 1:**
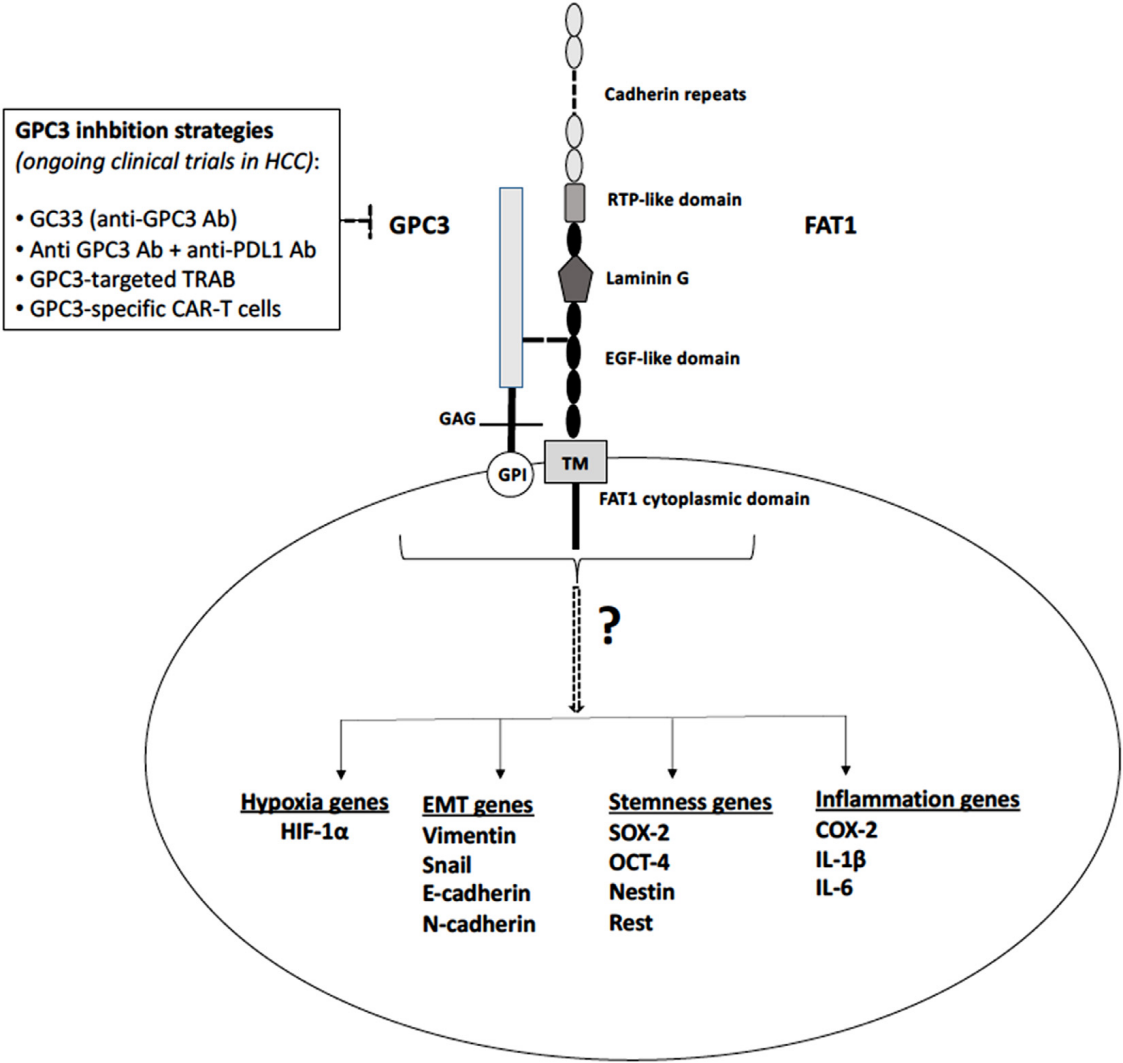
Schematic representation showing various tumor-related signaling pathways modulated by FAT1 in cancer cells, including glioma and hepato-cellular carcinoma (HCC) cells. However, it remains to be analysed in detail whether activation of these pathways is an outcome of a functional GPC3-FAT1 complex. Future studies are required to determine whether GPC3 (or other isoforms of glypicans depending on tissue-type) interacts with FAT1 to induce these signaling events as well as investigate the missing molecular links between increased FAT1 expression and activation of these pathways in cancer cells. It might be possible in the future to use available GPC3 inhibitors in patients stratified by upregulated FAT1 and downstream genes, although the same entails further research. Abbreviations: GPC3, glypican-3; TM, transmembrane domain; GAG, glycosaminoglycan; GPI, glycosylphosphatidylinositol; RTP, receptor tyrosine phosphatase; EGF, epidermal growth factor; Ab, antibody; TRAB, T cell-redirecting antibody; CAR-T cells, chimeric antigen receptor T cells.

FAT1 cytoplasmic domain has been shown to bind with β-catenin, whereas mutated FAT1 is unable to sequester β-catenin at the cell membrane, thereby potentiating transcriptional activity of β-catenin and Wnt pathway activation [3]. Comparison of FAT1 with another atypical cadherin MUCDHL *M* which also mediates interaction with β-catenin via its cytoplasmic tail [9] suggests implications of interaction of FAT1 with an extracellular binding partner. It was found that MUCDHL *M* deleted of its extracellular domain, while still retaining intact transmembrane and cytoplasmic domains, was unable to co-immunoprecipitate β-catenin. This was found to result from an incompatible conformation of the cytoplasmic tail of MUCDHL M in the absence of extracellular domain. The same was verified by the use of a fusion protein containing extracellular region derived from interleukin-2 receptor α subunit (IL2R) which was able to restore β-catenin binding by effectively reinstating the compatible conformation of the cytoplasmic segment [9]. Hence, in the new light of GPC3 as an interacting partner, it might be interesting to investigate whether its binding to FAT1 extracellular domain modulates β-catenin sequestration or alters its transcriptional activity, by inducing FAT1 cytoplasmic domain to adopt or deform a compatible conformation; and subsequently determines the fate of Wnt pathway.

Clinical utility of extracellular FAT1 domain has been established in pancreatic adenocarcinoma where soluble FAT1 ectodomain was found to act as serum biomarker [10]. An expression analysis of isoforms of glypicans in pancreatic cancer cells followed by co-IP analysis might reveal whether GPC-FAT1 interaction is necessary for causing conformation changes that might facilitate extracellular FAT1 cleavage.

While FAT1 is still a new candidate in cancer research, GPC3 has been investigated extensively as prognostic biomarker and therapeutic target. The significance of serum GPC3 levels and GPC3 immunoreactivity has been well-discussed in HCC patients [11]. Ongoing clinical trials include the use of GC33 (codrituzumab), an anti-GPC3 antibody which has been found to debilitate cancer cells via antibody-dependent cellular cytotoxicity and/or complement-dependent cytotoxicity and has shown prognostic merit for HCC patients in phase II trials. Moreover, a phase I clinical trial that combines anti-GPC3 antibody with immune checkpoint inhibitors (anti-PD-L1 antibodies) has shown well-tolerated immune response and potential anti-tumor activity in GPC3-overexpressing HCC. A recently developed bifunctional antibody named GPC3-targeted TRAB (T cell-redirecting antibody) has shown immunotherapeutic promise even in non-HCC cancers that express low but distinct levels of GPC3 and is currently in phase I trial. Furthermore, GPC3 serves as a promising cell surface antigen for generation of CAR-T cells against HCC cells and few clinical trials have been registered. Thus, GPC3 inhibitors facilitate a chance to abrogate FAT1-mediated oncogenic signaling, if future studies in GPC3-expressing tumors provide strengthening evidence of GPC3 acting as upstream initiator or regulator of FAT1 signaling event.

Thus, the study by Meng et al. warrants a screening of GPC3-FAT1 interaction in diverse cancer cell lines other than HCC. Further in-depth analysis may shed light on the possibility of a mechanistic role of GPC3-FAT1 binding in triggering FAT1-regulated pathways in various cancers. Needless to say, the findings presented in the highlighted study may be extended to patient-derived cultures to perform co-localization and co-IP analyses; and finally, to *in-vivo* mice studies. The study definitely exposes newer points to curtail FAT1-modulated oncogenic pathways in HCC by use of potential GPC3 inhibitors, which might be applicable to other tumors.

## CRediT authorship contribution statement

**Khushboo Irshad:** Conceptualization, Writing - review & editing. **Nargis Malik:** Writing - review & editing. **Manvi Arora:** Writing - review & editing. **Yakhlesh Gupta:** Writing - review & editing. **Subrata Sinha:** Conceptualization, Writing - review & editing. **Kunzang Chosdol:** Conceptualization, Writing - review & editing.

## Declaration of Competing Interest

The authors declare that they have no competing interests.
